# Comparison of hemoglobin variability between short- and intermediate-acting erythropoiesis-stimulating agents: a retrospective cohort study

**DOI:** 10.12701/jyms.2026.43.36

**Published:** 2026-05-28

**Authors:** Da Eun Park, Hae Dong Choi, Young Eun Park, Ki Baek Kim, A Young Kim, Kyu Hyang Cho, Jong Won Park, Jun Young Do, Seok Hui Kang

**Affiliations:** Division of Nephrology, Department of Internal Medicine, Yeungnam University College of Medicine, Daegu, Korea

**Keywords:** Anemia, Anemia management, Erythropoiesis-stimulating agents, Hemoglobin, Renal dialysis

## Abstract

**Background:**

This study aims to compare the effects of short- and intermediate-acting erythropoiesis-stimulating agents (ESAs) on hemoglobin (Hb) variability in patients undergoing maintenance hemodialysis.

**Methods:**

This retrospective cohort study included 119 patients who were classified into two groups based on ESA type: short-acting ESA (epoetin alfa/beta [EPO], n=48) and intermediate-acting ESA (darbepoetin alfa [DPO], n=71). Hb levels were measured 11 times at 4-week intervals from ESA therapy initiation to 40 weeks of follow-up. This study used established metrics from previous research, including standard deviation (SD), coefficient of variation (CV), and residual SD, to quantify Hb variability.

**Results:**

Over the 40-week study period with 4-week measurement intervals, mean Hb levels were comparable between the two ESA groups. In the EPO and DPO groups, SD was 0.67±0.19 g/dL and 0.69±0.22 g/dL, respectively (*p*=0.516), CV was 0.06±0.02 and 0.07±0.02, respectively (*p*=0.480), and residual SD was 0.73±0.21 g/dL and 0.76±0.26 g/dL, respectively (*p*=0.463). No significant differences in Hb variability were observed between the groups over 40 weeks using SD, CV, and residual SD.

**Conclusion:**

This retrospective cohort study showed comparable mean Hb levels and Hb variability indices between groups. With respect to Hb variability, the findings reveal no clear basis for prioritizing either short-acting or intermediate-acting ESA.

## Introduction

Chronic kidney disease (CKD) progression impairs renal function, triggering diminished erythropoietin synthesis and disrupting iron homeostasis, thereby establishing anemia as a cardinal clinical manifestation of CKD [1]. Erythropoietin deficiency suppresses erythroid activity in the bone marrow, causing anemia that amplifies cardiovascular risk and worsens the overall prognosis [2]. Nonetheless, erythropoiesis-stimulating agents (ESAs) have emerged as a primary strategy for correcting anemia and maintaining hemoglobin (Hb) levels within therapeutic targets in patients with CKD.

The two principal ESAs currently used in clinical practice are short-acting (i.e., epoetin alfa/beta [EPO]) and intermediate-acting (i.e., darbepoetin alfa [DPO]) ESAs. Although both these agents promote erythropoiesis, they differ substantially in their structural properties, pharmacokinetic profiles, and biological activities [3]. The subcutaneous administration of intermediate-acting ESA yields a half-life of approximately 48.8 hours, a threefold increase compared to that of short-acting ESA, theoretically allowing less frequent dosing and enhanced treatment adherence [3]. However, direct comparative evidence of superior Hb stability remains limited. Hb variability has emerged as a novel prognostic indicator associated with cardiovascular events, hospitalization rates, and mortality in anemia management [4]. Therefore, elucidating the differences in Hb variability between ESAs in patients with CKD may be clinically significant.

Recently, newer therapeutic options for CKD-related anemia, including hypoxia-inducible factor prolyl hydroxylase inhibitors (HIF-PHIs; e.g., roxadustat and daprodustat) and long-acting ESAs, such as continuous erythropoietin receptor activators, have been introduced into clinical practice. These agents offer potential advantages in terms of dosing intervals, oral administration, and erythropoietic regulation. Despite these advances, short- and intermediate-acting ESAs remain the most used first-line therapies in many hemodialysis centers worldwide. Additionally, the real-world use of HIF-PHIs continues to be influenced by factors such as costs, reimbursement policies, prescription restrictions, and limited long-term safety data in certain populations. Therefore, the selection between EPO and DPO remains a clinically relevant issue in contemporary anemia management for patients undergoing maintenance hemodialysis.

Therefore, this study aims to compare the effects of short- and intermediate-acting ESAs on Hb variability in patients undergoing maintenance hemodialysis. By analyzing longitudinal Hb trends and multiple variability indices across individual patients, we evaluated whether ESA selection influences the overall stability of anemia management in real-world clinical practice.

## Methods

**Ethics statement:** This study was approved by the Institutional Review Board (IRB) of Yeungnam University Hospital (IRB No: YUMC-2026-03-010). The requirement for informed consent was waived due to the retrospective design of the study. All the procedures were conducted in accordance with the ethical standards of the Declaration of Helsinki.

### 1. Study population

**Fig. 1.**
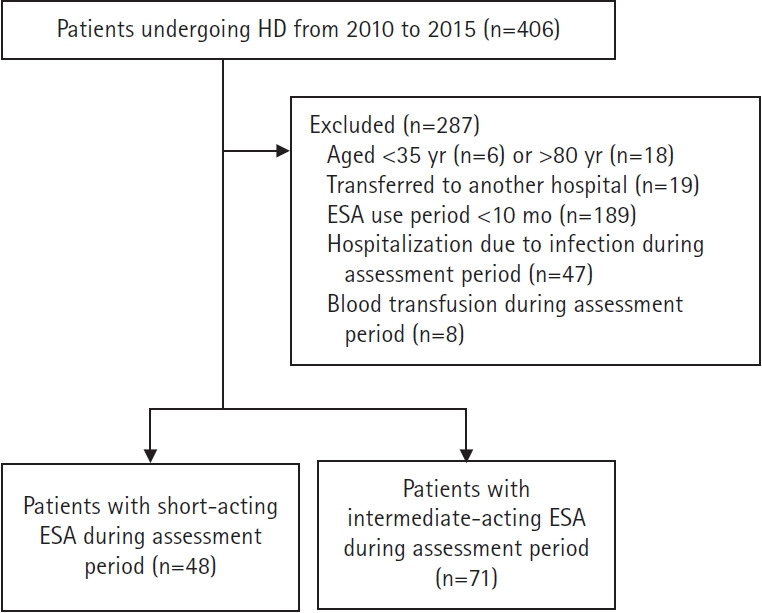
Flow diagram of patient selection and allocation to short- and intermediate-acting ESA groups. HD, hemodialysis; ESA, erythropoiesis-stimulating agent.

This retrospective cohort study included 406 patients who underwent maintenance hemodialysis at the hemodialysis unit of Yeungnam University Hospital between January 2010 and December 2015 (Fig. 1). We excluded patients aged <35 years (n=6) and >80 years (n=18), those transferred to another facility with discontinuation of hemodialysis (n=19), and those receiving ESA for <40 weeks, including those with cross-use of EPO and DPO (n=189). Additional exclusion criteria included hospitalization due to infection (n=47) and red blood cell transfusion within 1 month before enrollment or during the study period (n=8). All participants underwent continuous hemodialysis for at least 40 weeks and received EPO or DPO. Ultimately, 119 patients were included and classified into two groups based on ESA type: short-acting ESA (EPO, n=48) and intermediate-acting ESA (DPO, n=71). In this study, all 48 patients in the short-acting ESA group were administered epoetin beta (Recormon; F. Hoffmann-La Roche, Basel, Switzerland), and none were administered epoetin alfa. All 71 patients in the intermediate-acting ESA group received DPO (Nesp; Kyowa Kirin Co., Tokyo, Japan). According to the standardized anemia management protocol at our institution, all patients in both groups received intravenous ESA therapy exclusively during the study period; none of the drugs were administered subcutaneously.

### 2. Study variables

This retrospective cohort study included clinical and biochemical parameters to evaluate Hb variability based on the ESA type. Hb levels were measured 11 times at 4-week intervals from ESA therapy initiation until 40 weeks of follow-up. The baseline Hb level was defined as the value measured immediately before the first ESA administration. Before ESA initiation, baseline laboratory and clinical parameters, including serum C-reactive protein (CRP, mg/dL), albumin (g/dL), serum iron (μg/dL), ferritin (ng/mL), total iron-binding capacity (μg/dL), transferrin saturation (%), dialysis adequacy (Kt/V_urea_), ultrafiltration volume (UFV, L/session), and body mass index (kg/m^2^), were assessed.

Concomitant medication use, including oral or intravenous iron supplementation, renin-angiotensin system blockers, aspirin, clopidogrel, other antiplatelet agents, anticoagulants, and statins, was recorded to evaluate potential effects. Other antiplatelet agents, including cilostazol, sarpogrelate, beraprost, and naftazone, were defined as oral antiplatelet drugs other than aspirin and clopidogrel. Anticoagulants included warfarin and non-vitamin K oral anticoagulants. The Charlson Comorbidity Index was calculated for all patients to assess the comorbidity burden, including the presence of diabetes mellitus, myocardial infarction, congestive heart failure, cerebrovascular disease, peptic ulcer disease, liver disease, solid tumors, and hematologic disorders [5].

Owing to the lack of a consensus definition of Hb variability in the literature, this study used established metrics from previous research, including standard deviation (SD), coefficient of variation (CV), and residual SD to quantify Hb variability [6,7]. SD was calculated as follows: SD=√[Σ (Hbi−Hb_mean_)²/(n−1)], and CV as CV=(SD/Hb_mean_)×100%. The residual SD was defined as the SD of the residuals from the individual linear regression line fitted to serial Hb measurements for each patient [4,8]. These indices were used to compare and analyze the Hb fluctuation patterns between the two groups.

Given that ESA dosing units differ between EPO and DPO, doses of DPO were converted to ESA-equivalent units using a fixed intravenous conversion ratio of 1 μg DPO to 200 international units (IU) EPO for standardization [9]. The converted mean ESA doses were included in the baseline characteristic comparisons and applied consistently in the subsequent subgroup analyses. All ESA administrations were performed exclusively via the intravenous route, with no subcutaneous administration.

### 3. Statistical analysis

Clinical and biochemical variables obtained at the initiation of ESA treatment were used to compare baseline characteristics between the short- and intermediate-acting ESA groups. All the statistical analyses were performed with IBM SPSS ver. 29 (IBM Corp., Armonk, NY, USA). Continuous variables were assessed for normality and compared using independent samples t-tests, whereas categorical variables were compared using chi-square tests. The *p*-values were calculated for each variable to determine statistical significance. Hb levels were measured 11 times at 4-week intervals after the initiation of ESA therapy. At each time point, the mean, SD, and 95% confidence interval (CI) for the Hb level were calculated. Differences in Hb trends over time between the groups were analyzed using repeated-measures analysis of variance. Hb variability in individual patients was quantified using three indices: SD, CV, and residual SD. The residual SD was calculated as the SD of the residuals from the individual linear regression line fitted to serial Hb measurements for each patient. Variability indices were compared between the groups using independent samples t-tests. Subgroup analyses based on sex, age (≥65 years), UFV (median split), and ESA dosage (median split) were performed using the same statistical methods.

Using the observed sample sizes (EPO group, n=48; DPO group, n=71) and the pooled SD for each parameter, a post-hoc power analysis was performed to estimate the minimum detectable difference (MDD) at 80% statistical power using the following standard formula:


$$\mathrm{MDD}=\left(Z_{1-\alpha /2}+Z_{1-\beta }\right)\times \sigma \times \sqrt{\frac{1}{n1}+\frac{1}{n2}},$$

where α represents the significance level, 1-β indicates the statistical power, σ denotes the pooled SD of the two groups, and *n1* and *n2* represent the respective sample sizes of the groups. Statistical significance was defined as *p*<0.05.

## Results

### 1. Baseline characteristics

Table 1 presents the baseline characteristics of the 119 patients. The mean age in the EPO and DPO groups was 63.8±12.3 and 59.7±10.2 years, respectively (*p*=0.052). Both groups were generally well balanced at baseline, with no significant differences in age, sex distribution (predominantly male), body mass index, diabetes status, Charlson Comorbidity Index score, or laboratory parameters including Hb level, iron indices, and dialysis adequacy. However, UFV was statistically higher in the DPO group (2.1±1.0 L/session) than in the EPO group (1.5±1.0 L/session, *p*=0.004). Concomitant medication use was similar between the groups, with no significant differences in the proportion of patients receiving oral iron supplementation, renin-angiotensin system blockers, aspirin, clopidogrel, other antiplatelet agents, anticoagulants, or statins. CRP levels were 0.558±0.603 mg/dL in the EPO group and 0.591±0.772 mg/dL in the DPO group (*p*=0.792). Serum albumin levels were 3.866±0.519 g/dL and 3.862±0.442 g/dL in the EPO and DPO groups, respectively (*p*=0.968). No statistically significant differences were observed between the EPO and DPO groups in either parameter.

Additionally, we evaluated the use of intravenous iron during the study period and incorporated the corresponding data into Table 1. Venoferrum 100 mg was the only intravenous iron preparation administered to this cohort. Only a small proportion of patients underwent intravenous iron therapy: three patients (6.3%) in the EPO group and one patient (1.4%) in the DPO group. No statistically significant difference was observed in intravenous iron use between the groups (*p*=0.305).

### 2. Comparison of hemoglobin variability based on erythropoiesis-stimulating agent type

**Fig. 2.**
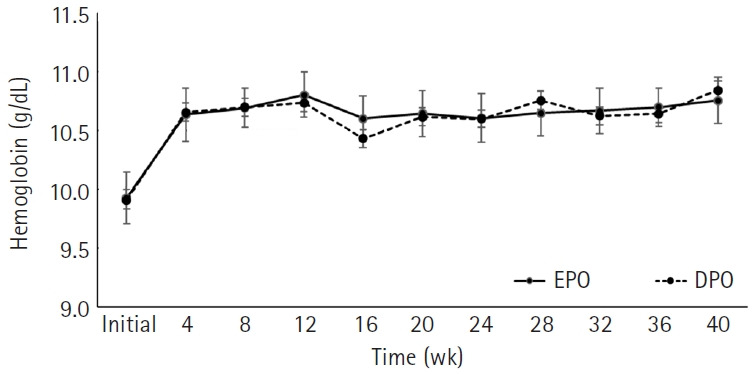
Temporal trends in hemoglobin levels according to ESA type. Data are presented as mean hemoglobin levels with 95% confidence intervals. ESA, erythropoiesis-stimulating agent; EPO, epoetin alfa/beta; DPO, darbepoetin alfa.

Over the 40-week study period with 4-week measurement intervals, the mean Hb levels were comparable between the two ESA groups. No statistically significant differences in the mean Hb concentrations were observed between the groups at any time point (Fig. 2, Table 2).

**Fig. 3.**
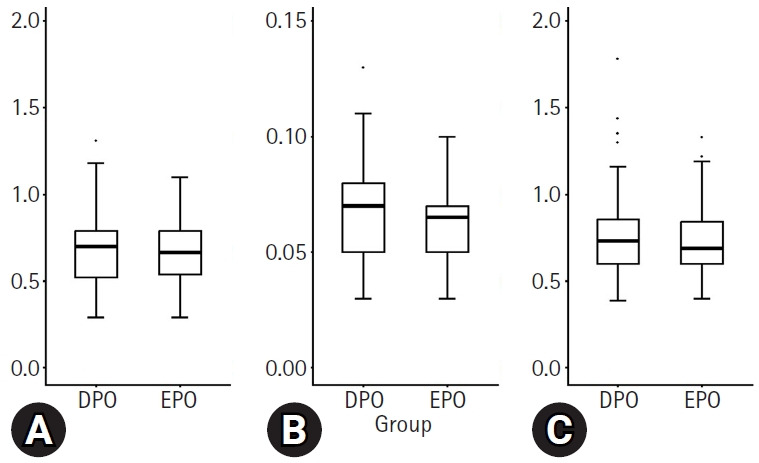
Boxplots of hemoglobin variability indices in the EPO and DPO groups. Boxplots depict the distribution of (A) standard deviation (SD), (B) coefficient of variation (CV), and (C) residual SD. The p-values from the independent samples t-tests were 0.516 for SD, 0.480 for CV, and 0.463 for residual SD, indicating no significant between-group differences (all p>0.05). EPO, epoetin alfa/beta; DPO, darbepoetin alfa.

In the overall cohort, Hb variability was assessed using SD, CV, and residual SD (Fig. 3). In the EPO and DPO groups, the SD was 0.67±0.19 g/dL and 0.69±0.22 g/dL, respectively (*p*=0.516), the CV was 0.06±0.02 and 0.07±0.02, respectively (*p*=0.480), and the residual SD was 0.73±0.21 g/dL and 0.76±0.26 g/dL, respectively (*p*=0.463). No significant differences in these Hb variability parameters were observed between the groups over 40 weeks. The box plots in Fig. 3 show substantial overlap in the distributions of all three variability indices between the EPO and DPO groups, suggesting minimal between-group differences in Hb variability.

The calculated MDDs were 0.36 g/dL for SD, 0.03 for CV, and 0.11 g/dL for residual SD. The post-hoc power analysis revealed that the study was sufficiently powered to detect moderate clinically meaningful differences in Hb variability indices, suggesting that the absence of statistically significant differences was unlikely to be solely attributable to an insufficient sample size.

### 3. Subgroup analyses

Subgroup analyses stratified by sex, age (≥65 years), UFV (2 L/session, median split), and ESA dosage (7,450 IU/week, median split) revealed no statistically significant differences between the ESA groups across the three Hb variability indices (SD, CV, and residual SD) (Table 3). However, within the high ESA dose subgroup, the residual SD was slightly greater in the DPO group than in the EPO group (*p*=0.049).

## Discussion

In this retrospective cohort study of patients undergoing maintenance hemodialysis, longitudinal Hb trajectories and variability were compared between the EPO and DPO groups. Both groups exhibited comparable Hb levels and trends, along with broadly similar Hb variability as assessed by SD, CV, and residual SD over 40 weeks, with no statistically significant differences between the groups. However, in the high ESA dose subgroup, the Hb variability based on residual SD was marginally higher in the DPO group than in the EPO group.

Several studies have compared short- and intermediate-acting ESAs in patients undergoing hemodialysis. In a randomized, open-label, non-inferiority phase III trial on Chinese patients undergoing hemodialysis, the change in Hb from baseline was −0.07 g/dL in the darbepoetin-switch group and −0.15 g/dL in the EPO-continuation group. This yielded a between-group difference of 0.08 g/dL (95% CI, −0.22 to 0.39) within the non-inferiority margin of −1.0 g/dL, and target Hb maintenance rates did not differ significantly (*p*=0.81) [10]. In a meta-analysis of patients undergoing dialysis, the overall mean Hb was 11.5 g/dL (95% CI, 11.3–11.7) with an SD of 0.99 g/dL (95% CI, 0.88–1.09). No statistically significant differences were observed between short- and intermediate-acting ESAs in mean Hb, Hb variability, or ESA dose variability [11].

Hb variability in patients undergoing hemodialysis reflects a complex interplay of patient- and treatment-related factors, including ESA dose and dosing frequency, iron status and supplementation, inflammation, intercurrent events (such as infection or hospitalization), occult hemorrhage, and comorbid conditions [12,13]. Additionally, more frequent ESA dose adjustments and clinical instability are associated with greater intraindividual Hb fluctuations, whereas relatively stable dosing and fewer intercurrent events are associated with lower variability [13]. In this context, DPO has a half-life approximately three times longer than that of EPO, enabling less frequent and more consistent dosing. Theoretically, this may reduce rapid peaks and troughs during erythropoietic stimulation, thereby attenuating long-term Hb variability [3,4,14]. However, in Korea, where Hb levels are closely monitored and ESA dosing follows standardized protocols, rapid dose modification in response to Hb fluctuations is feasible. Under these circumstances, the shorter half-life of EPO may not lead to significantly greater Hb variability, potentially accounting for the lack of significant differences between EPO and DPO in the present study.

Previous large-scale studies have shown Hb SD values of approximately 0.9 to 1.0 g/dL in patients undergoing hemodialysis [11,15]. However, in the present study, the observed SD values were lower (0.67–0.69 g/dL). This finding may reflect the relatively stable clinical setting of our cohort, as this was a single-center study conducted using a standardized ESA management protocol. In addition, patients with conditions that could substantially increase Hb variability, including active infection, blood transfusion, hospitalization, and crossover ESA use, were excluded. To determine whether the study sample size was sufficient to detect clinically meaningful differences, a post-hoc power analysis was performed. Based on previous studies indicating that the overall magnitude of Hb variability in patients undergoing hemodialysis was approximately 1.0 g/dL and considering a moderate effect size according to Cohen’s criteria (d of approximately 0.5), we considered a between-group difference of approximately 0.3 to 0.4 g/dL in Hb SD to be clinically meaningful. The calculated MDD for SD was approximately 0.36 g/dL, indicating that this study was adequately powered to detect moderate clinically relevant differences in Hb variability. The observed between-group differences were substantially smaller than this threshold. The mean differences in SD, CV, and residual SD between the EPO and DPO groups were approximately 0.01 to 0.03 and were not statistically significant. Therefore, our findings suggest not only the absence of statistical significance but also that the practical and clinical differences in Hb variability between EPO and DPO treatments are likely minimal under relatively stable Hb management conditions.

This study has several strengths compared with previous ones. First, unlike studies that used a single metric such as SD or CV to assess Hb variability, SD, CV, and residual SD were evaluated simultaneously in this study for a more comprehensive assessment [4,15]. In particular, residual SD, which reflects variability after the removal of temporal trends, is less influenced by ESA dose titration or gradual shifts, and thus better captures intrinsic Hb variability [6]. Second, the present study was conducted in a single-center hemodialysis cohort from an Asian country with consistent anemia management protocols, Hb targets, and dialysis prescriptions. This design reduces the inter-center variability typical of multicenter or multinational cohorts and enables a more controlled assessment of the effect of ESA type on Hb variability. However, these findings may have limited generalizability to other countries and ethnic groups. Third, although a 40-week observation period does not strictly constitute a long-term follow-up, it exceeds the 3- to 6-month duration of many clinical trials and short-term switch studies. This timeframe represents a medium-term real-world period suitable for evaluating repeated ESA dose adjustments and Hb fluctuation patterns in routine clinical practice.

None of the ESAs were administered subcutaneously. Therefore, no between-group differences in the ESA administration route were observed, and a route-stratified sensitivity analysis was not applicable. Ibbotson et al. reported that the pharmacokinetic profiles of ESAs differ substantially according to the ESA type and administration route [3]. Specifically, DPO exhibits a substantially longer terminal half-life than EPO following intravenous and subcutaneous administration (approximately 25.3 vs. 8.5 and 48.8 vs. 19.4–24.2 hours for the intravenous and subcutaneous routes, respectively), which could theoretically result in more sustained erythropoietic stimulation and reduced Hb variability. However, despite these pharmacokinetic differences, the Hb variability indices (SD, CV, and residual SD) were similar between the two groups. These findings suggest that, under a standardized intravenous-based ESA dose-adjustment protocol, the longer half-life of DPO may not translate into clinically meaningful differences in Hb variability in patients who are stable and undergoing hemodialysis.

A recent nationwide registry study by Kang and Kim [16] analyzed 48,726 patients undergoing hemodialysis and indicated statistically significant differences in Hb variability according to ESA type. The results showed that residual SD was lowest in the short-acting ESA group compared to the values in the intermediate- and long-acting ESA groups (short, 0.60 g/dL; intermediate, 0.68 g/dL; and long, 0.64 g/dL). Conversely, this single-center study showed similar Hb variability between the EPO and DPO groups, with minimal differences in the SD, CV, and residual SD indices. Several factors could explain this discrepancy. First, the study by Kang and Kim [16] was based on a nationwide registry reflecting heterogeneous real-world practice patterns, including differences in ESA dose-adjustment strategies, Hb target ranges, physician preferences, and center-specific anemia management protocols. In contrast, our study was conducted at a single institution using a highly standardized ESA titration protocol and consistent Hb target management, which may have reduced the overall Hb variability and minimized the between-group differences. Second, differences in Hb monitoring methodologies may also have contributed. The nationwide registry study evaluated monthly Hb measurements over 6 months, whereas our study analyzed serial Hb measurements obtained at 4-week intervals over 40 weeks, allowing for a more continuous longitudinal assessment of Hb variability within individual patients. Importantly, the absolute differences in residual SD reported in the nationwide registry study were relatively modest despite achieving statistical significance. Therefore, rather than contradicting the findings of Kang and Kim, our findings may provide complementary evidence indicating that under well-standardized anemia management protocols, the practical differences in Hb variability between the EPO and DPO groups may become clinically minimal in patients who are stable and undergoing maintenance hemodialysis.

We acknowledge that this study was conducted using data collected between 2010 and 2015, during which anemia management was primarily guided by the 2012 Kidney Disease: Improving Global Outcomes (KDIGO) recommendations, with target Hb levels generally maintained within the range of 10 g/dL to 11 g/dL. In contrast, the updated 2026 KDIGO guidelines recommend initiating ESA therapy at Hb levels of ≤9 to 10 g/dL and <11.5 g/dL during ESA maintenance therapy [17]. Therefore, differences in the current Hb target strategies should be considered when interpreting the absolute Hb levels and variability observed in this study. However, the EPO and DPO groups were managed using the same institutional anemia management protocol and identical Hb target ranges. Accordingly, although historical Hb targets may limit the direct generalizability of the findings to current practice environments, they are unlikely to have substantially affected the relative comparison of Hb variability between the two ESA groups. Additionally, as newer therapeutic options such as HIF-PHIs and long-acting ESAs have become increasingly incorporated into clinical practice, further studies are needed to evaluate Hb variability under contemporary anemia management strategies.

The conversion ratio of 1 μg DPO=200 IU EPO used in this study was based on the initial manufacturer-recommended conversion strategy and previous studies, including that by Arrieta et al. [9]. Although Korean and international clinical studies have used conversion ratios of 1 μg DPO=200 to 250 IU EPO, these values may vary depending on the patient population, treatment response, route of administration, and clinical practice patterns. However, because the primary aim of the present study was to compare longitudinal Hb variability rather than establish precise dose equivalence between ESA formulations, we uniformly applied a fixed conversion ratio across all patients to ensure consistent ESA dose normalization throughout the study period. We believe that using a single standardized conversion factor minimized potential bias in the comparative analysis of Hb variability between the ESA groups. In addition, all ESA administrations in this study were performed exclusively via the intravenous route, with no subcutaneous administration. Therefore, the conversion ratio should be interpreted in the context of intravenous administration. Furthermore, we recalculated ESA-equivalent doses using a 1:250 conversion ratio. Under this alternative conversion method, the equivalent ESA dose in the DPO group was 9,865±7,425 IU/week, compared to 8,176±2,711 IU/week in the EPO group, and the between-group difference was not statistically significant (*p*=0.083). These findings suggest that the overall interpretation of the study results was not affected by the selected conversion ratio.

In patients receiving a high ESA dose, Hb variability as assessed by residual SD was marginally higher in the DPO group than in the EPO group. As this was a retrospective study, determining the underlying mechanism can be challenging, and the findings may reflect chance statistical significance due to multiple comparisons. However, in patients requiring high ESA doses, short-acting agents may permit a more rapid adjustment to changes in Hb levels because of their shorter duration of action. In contrast, intermediate-acting agents may have prolonged residual effects, limiting rapid dose adjustment and potentially contributing to abrupt declines in Hb levels. The observed differences in residual SD among patients with higher ESA requirements may partly reflect the limitations of the fixed dose-conversion approach rather than the direct pharmacological effect of the ESA type alone. We acknowledge that the dose conversion between EPO and DPO is not strictly linear in real-world clinical practice. In this study, we applied a fixed conversion ratio of 1 μg DPO=200 IU EPO for dose normalization. However, the conversion ratios in studies may vary according to the baseline ESA requirement, with higher ratios (often 250:1–300:1 or greater) being required in patients treated with higher epoetin doses.

To address this issue, we also evaluated the sensitivity of baseline ESA dose comparisons using alternative conversion ratios. When conversion ratios of 1:250 and 1:300 were applied, the *p*-values for the baseline ESA dose comparisons between the two groups were 0.083 and 0.002, respectively, indicating that the comparison was sensitive to the assumed conversion ratio. These findings indicate that the use of a fixed 1:200 conversion ratio may have led to an underestimation of the erythropoietin-equivalent dose in patients requiring higher ESA doses. Nevertheless, the overall pattern of Hb variability indices was not materially altered across conversion assumptions; therefore, the main conclusion that Hb variability was broadly similar between the EPO and DPO groups under a standardized anemia management protocol remains valid.

This study had some limitations. First, as this was a retrospective, single-center study with a relatively small hemodialysis cohort, causal inference is inherently limited, and generalizability may be restricted. We acknowledge that the present study may have been underpowered to detect small differences in Hb variability between the two groups. In the post-hoc power analysis, assuming a small effect size according to Cohen’s criteria (d=0.2), corresponding to an SD difference of approximately 0.14 g/dL, the estimated statistical power with the current sample sizes (EPO, n=48; DPO, n=71) was approximately 20% to 30%. Accordingly, minimal differences in Hb variability may not have been detected even if they truly existed, reflecting the possibility of a Type II error (β≈0.7). However, as discussed above, the study had sufficient power to detect moderate, clinically meaningful differences in Hb variability (–0.3 to 0.4 g/dL). Therefore, although the possibility of not detecting small differences cannot be excluded, the observed near-zero differences between the groups suggest that any true difference in Hb variability between the EPO and DPO groups is likely clinically minimal. Second, although we adjusted for major clinical variables that could affect Hb variability, residual confounding factors due to occult hemorrhage, inflammation, and comorbidities could not be excluded. Third, although multiple subgroup comparisons and variability analyses were conducted without formal correction for multiplicity, any isolated findings, such as the higher residual SD observed in the high-dose ESA subgroup of the DPO group, should be interpreted with caution [18].

In conclusion, this retrospective cohort study showed comparable mean Hb levels and Hb variability indices (SD, CV, and residual SD) between groups. These findings suggest that with respect to Hb variability, no clear basis exists for prioritizing either short-acting or intermediate-acting ESAs. However, given the inherent limitations of this study, further well-designed investigations are required to confirm these findings.

## Figures and Tables

**Table 1.** Baseline characteristics of the study population

Characteristic	EPO group	DPO group	*p*-value
No. of patients	48	71	
Age (yr)	63.8±12.3	59.7±10.2	0.052
Male sex	27 (56.3)	48 (67.6)	0.208
Body mass index (kg/m^2^)	22.1±2.7	22.8±2.8	0.147
Diabetes mellitus	26 (54.2)	39 (54.9)	0.935
CCI score	5.8±1.7	5.8±1.9	0.927
Kt/V_urea_	1.5±0.3	1.6±0.3	0.182
Ultrafiltration volume (L/session)	1.5±1.0	2.1±1.0	0.004
Hemoglobin (g/dL)	9.9±0.8	9.9±0.8	0.892
Serum iron (μg/dL)	72.1±87.8	57.8±33.1	0.215
Ferritin (ng/mL)	302.6±379.3	295.9±264.1	0.910
Total iron-binding capacity (μg/dL)	205.5±39.5	203.9±40.5	0.833
Transferrin saturation (%)	44.4±91.8	30.9±23.4	0.238
C-reactive protein (mg/dL)	0.558±0.603	0.591±0.772	0.792
Albumin (g/dL)	3.866±0.519	3.862±0.442	0.968
Use of ferrous sulfate	39 (81.3)	65 (91.5)	0.097
Use of intravenous iron	3 (6.3)	1 (1.4)	0.305
Use of RAS blocker	37 (77.1)	57 (80.3)	0.674
Use of aspirin	8 (16.7)	20 (28.2)	0.147
Use of clopidogrel	3 (6.3)	8 (11.3)	0.522
Use of other antiplatelet	5 (10.4)	6 (8.5)	0.755
Use of anticoagulant	7 (14.6)	9 (12.7)	0.765
Use of statin	8 (16.7)	18 (25.4)	0.261
ESA dose (IU/week)	8,176±2,711	7,892±5,940	0.726

Values are presented as number, mean±standard deviation, or number (%). The *p*-values were calculated using the independent samples t-test for continuous variables and the chi-square test for categorical variables. EPO, epoetin alfa/beta; DPO, darbepoetin alfa; CCI, Charlson Comorbidity Index; Kt/V_urea_, dialysis adequacy; RAS, renin-angiotensin system; ESA, erythropoiesis-stimulating agent; IU, international unit.

**Table 2.** Longitudinal trends in mean hemoglobin levels in the EPO vs. DPO groups

Time (week)	Hemoglobin level (g/dL)		*p*-value
	EPO group	DPO group	
0 (baseline)	9.9±0.8	9.9±0.8	0.892
4	10.6±0.8	10.7±0.8	0.888
8	10.7±0.6	10.7±0.7	0.965
12	10.8±0.7	10.7±0.8	0.631
16	10.6±0.7	10.4±0.7	0.183
20	10.6±0.7	10.6±0.8	0.837
24	10.6±0.7	10.6±0.7	0.966
28	10.7±0.7	10.8±0.7	0.412
32	10.7±0.7	10.6±0.7	0.747
36	10.7±0.6	10.6±0.6	0.619
40	10.8±0.7	10.8±0.6	0.473

Data are presented as mean±standard deviation. Group differences were analyzed using the independent samples t-test. EPO, epoetin alfa/beta; DPO, darbepoetin alfa.

**Table 3.** Subgroup analysis of hemoglobin variability by sex, age, UFV, and mean ESA dose in the EPO and DPO groups

Variable	SD (g/dL)			CV			Residual SD (g/dL)		
	EPO group	DPO group	*p*-value	EPO group	DPO group	*p*-value	EPO group	DPO group	*p*-value
Sex									
Male	0.69±0.21	0.67±0.18	0.654	0.06±0.02	0.06±0.02	0.668	0.76±0.25	0.73±0.22	0.618
Female	0.64±0.16	0.74±0.28	0.137	0.06±0.02	0.07±0.03	0.119	0.69±0.15	0.83±0.33	0.084
Age (yr)									
≥65	0.64±0.21	0.65±0.22	0.832	0.06±0.02	0.06±0.02	0.922	0.73±0.25	0.72±0.24	0.884
<65	0.69±0.17	0.71±0.22	0.637	0.06±0.02	0.07±0.02	0.520	0.73±0.17	0.79±0.27	0.356
UFV (L/session)									
≥2	0.64±0.18	0.67±0.24	0.339	0.06±0.02	0.07±0.02	0.322	0.68±0.17	0.78±0.28	0.163
<2	0.68±0.20	0.67±0.19	0.905	0.06±0.02	0.06±0.02	0.957	0.76±0.23	0.74±0.23	0.743
ESA dose									
High^a)^	0.70±0.18	0.79±0.23	0.092	0.07±0.02	0.08±0.02	0.066	0.75±0.19	0.89±0.32	0.049
Low^b)^	0.61±0.19	0.63±0.19	0.666	0.06±0.02	0.06±0.02	0.585	0.70±0.25	0.69±0.18	0.817

Values were expressed as mean±standard deviation. UFV, ultrafiltration volume; ESA, erythropoiesis-stimulating agent; EPO, epoetin alfa/beta; DPO, darbepoetin alfa; SD, standard deviation; CV, coefficient of variation.s

^a)^ESA dose >median, ^b)^ESA dose ≤median.
